# Role of microRNA 690 in Mediating Angiotensin II Effects on Inflammation and Endoplasmic Reticulum Stress

**DOI:** 10.3390/cells9061327

**Published:** 2020-05-26

**Authors:** Kalhara R. Menikdiwela, Latha Ramalingam, Mostafa M. Abbas, Halima Bensmail, Shane Scoggin, Nishan S. Kalupahana, Asha Palat, Preethi Gunaratne, Naima Moustaid-Moussa

**Affiliations:** 1 Department of Nutritional Sciences, Obesity Research Institute, Texas Tech University, Lubbock, TX 79409, USA; kalhara.menikdiwela@ttu.edu (K.R.M.); latha.ramalingam@ttu.edu (L.R.); shane.scoggin@ttu.edu (S.S.); nishan.kalupahana@gmail.com (N.S.K.); 2 Qatar Computing Research Institute, Hamad Bin Khalifa University, Doha 34110, Qatar; mostafa.mn.abbas@gmail.com (M.M.A.); hbensmail@hbku.edu.qa (H.B.); 3 Department of Imaging Science and Innovation, Geisinger Health System, Danville, PA 17822, USA; 4 Department of Physiology, University of Peradeniya, Peradeniya 20400, Sri Lanka; 5 Biology and Biochemistry, University of Houston, Houston, TX 77204, USA; palat.asha@gmail.com (A.P.); phgunara@central.uh.edu (P.G.)

**Keywords:** adipocytes, renin–angiotensin system (RAS), obesity, miR-690, endoplasmic reticulum (ER) stress, inflammation

## Abstract

Overactivation of the renin–angiotensin system (RAS) during obesity disrupts adipocyte metabolic homeostasis and induces endoplasmic reticulum (ER) stress and inflammation; however, underlying mechanisms are not well known. We propose that overexpression of angiotensinogen (Agt), the precursor protein of RAS in adipose tissue or treatment of adipocytes with Angiotensin II (Ang II), RAS bioactive hormone, alters specific microRNAs (miRNA), that target ER stress and inflammation leading to adipocyte dysfunction. Epididymal white adipose tissue (WAT) from B6 wild type (Wt) and transgenic male mice overexpressing Agt (Agt-Tg) in adipose tissue and adipocytes treated with Ang II were used. Small RNA sequencing and microarray in WAT identified differentially expressed miRNAs and genes, out of which miR-690 and mitogen-activated protein kinase kinase 3 (MAP2K3) were validated as significantly up- and down-regulated, respectively, in Agt-Tg, and in Ang II-treated adipocytes compared to respective controls. Additionally, the direct regulatory role of miR-690 on MAP2K3 was confirmed using mimic, inhibitors and dual-luciferase reporter assay. Downstream protein targets of MAP2K3 which include p38, NF-κB, IL-6 and CHOP were all reduced. These results indicate a critical post-transcriptional role for miR-690 in inflammation and ER stress. In conclusion, miR-690 plays a protective function and could be a useful target to reduce obesity.

## 1. Introduction

Obesity is a complex disease where excess fat accumulation results in metabolic disruption in adipocytes. This in turn leads to macrophage infiltration [1], which then induces pro-inflammatory cytokine/adipokine secretion. Angiotensin II (Ang II) is one of the several pro-inflammatory adipokines secreted by adipocytes and is elevated in obesity, as demonstrated in both in vivo and in vitro studies [2,3]. Ang II is the key bioactive component of the renin–angiotensin system (RAS) and is primarily involved in regulation of blood pressure and fluid balance. However, overactivation of RAS in adipose tissue interrupts adipose homeostasis, causing inflammation, insulin resistance and cell stresses including oxidative and endoplasmic reticulum (ER) stress [2,3]. Nevertheless, underlying molecular mechanism/s by which RAS overactivation contributes to obesity-associated co-morbidities are yet to be elucidated.

MicroRNAs (miRNA) could be a possible mechanism by which RAS regulates molecular signaling pathways. They are small, evolutionarily conserved, non-coding RNA molecules approximately 22 nucleotides in size [4,5]. They play a pivotal role in regulating genes at post-transcriptional level either by cleaving messenger RNA (mRNA), or by translational repression or mRNA decay [6,7,8,9]. miRNA–mRNA interaction is broad and complex as a single miRNA could regulate hundreds of genes and each mRNA may interact with several miRNAs [5,10,11]. Impairments in miRNA regulation could be directly linked to diseases including obesity. Altered expression of miRNAs such as miR-143, miR-708 and miR-132 during obesity induces adipogenesis, ER stress and inflammation [12,13,14]. Although there are several studies performed to recognize RAS–miRNA association [15,16,17,18], the effect of RAS overactivation on miRNA expression in the adipose tissue during obesity remains to be explored. Identifying such miRNAs and new druggable targets from the miR-690-regulated gene networks would be a novel approach to manage RAS-mediated detrimental effects and could be used as an effective therapeutic solution to alleviate obesity.

In vivo and in vitro studies show that RAS-activated inflammation and cell stresses are partly mediated by mitogen-activated protein kinases (MAPKs) [19,20,21,22]. One of the MAPK pathways, p38MAPK, plays a crucial role in activating several downstream inflammatory cytokines where activation of p38 MAPK directly stimulates inflammatory markers such as interleukin-1 (IL-1), IL-6, IL-12, tumor necrosis factor α (TNFα) and NF-κB and also induces cellular stresses [22,23,24,25]. Several lines of evidence have shown that Ang II is involved in chronic disease progression by activating MAPKs (e.g., MAP3K7 and MAPK14/p38) [25,26,27]. Identifying miRNAs which directly target MAPKs could alleviate RAS-p38MAPKs-associated inflammation and cell stress.

Here we hypothesize that when RAS is overexpressed, miR-690 plays a protective role by targeting MAPKs, thereby reducing downstream pathways including inflammation and ER stress. We used epididymal white adipose tissue (WAT) from mice overexpressing angiotensinogen (Agt) specifically in the adipose tissue to identify affected miRNAs. We performed microRNA-Seq to identify differentially expressed miRNAs and identified miR-690 as the top candidate, based on its potential ability to regulate genes in inflammatory and cell stress pathways. miR-690 has a role in myeloid cell and osteogenic differentiation, but its role in RAS induced obesity is not yet known [28,29]. We demonstrated for the first time that miR-690 regulates inflammation and ER stress by directly targeting MAP2K3 when RAS is overexpressed in the adipose tissue. These findings confirm a probable protective role of miR-690 in regulating inflammation and ER stress in adipocytes.

## 2. Materials and Methods

### 2.1. Mouse Study

We used epididymal WAT from male wild type (Wt) C57BL6/J and angiotensinogen transgenic (Agt Tg) mice where the Agt gene was specifically overexpressed in the adipose tissue. These mice were fed a low fat (LF) diet with 10% kcal from fat for 12 weeks [2]. WAT was collected after euthanasia (CO_2_ inhalation) from mice and snap-frozen immediately in liquid nitrogen and stored at −80 °C for further analyses. The above study was approved by the University of Tennessee, institutional animal care and use committee (IACUC Protocol # 678 approved by TN: 1/6/2009 renewed every 3 years through 2013), and adipose tissue collected from these mice was analyzed at Texas Tech University.

### 2.2. Cell Cultures

Mouse pre-adipocytes 3T3-L1 cells were purchased from American Type Culture Collection (ATCC) (Manassas, VA, USA) and maintained at 37 °C in Dulbecco’s Modified Eagle’s Medium (DMEM) accompanied with fetal bovine serum (FBS 10%) (Thermo Fisher Scientific, Waltham, MA, USA). Media were also supplemented with antibiotics (50 g/mL streptomycin and 50 U/mL penicillin; Thermo Fisher Scientific, Waltham, MA, USA). 3T3-L1 pre-adipocytes were differentiated as previously described [3]. Differentiated 3T3-L1 adipocytes were treated with 10 nM Ang II (Sigma-Aldrich, St. Louis, MO, USA) [3], Ang II receptor blocker Telmisartan 1 mg/mL (Sigma-Aldrich, St. Louis, MO, USA). After 24 h of treatment, media and cells (3T3-L1 cells) were collected and stored at −80 °C for further analyses.

### 2.3. RNA and MicroRNA Isolation

Isolation of RNA was performed using RNeasy mini kit (Qiagen, Valencia, CA, USA). cDNA was reverse transcribed using Maxima reverse transcriptase (Thermo Fisher Scientific, Waltham, MA, USA) followed by gene expression by real-time quantitative polymerase chain reaction (RT-qPCR) using Sybr green master mix (Thermo Fisher Scientific, Waltham, MA, USA). Gene expression of individual genes was normalized to housekeeping genes 18S ribosomal RNA or GAPDH (List of primers Tables S1 and S2).

TaqMan™ Advanced miRNA cDNA Synthesis Kit (Thermo Fisher Scientific, Waltham, MA, USA) was used to reverse-transcribe cDNA from RNA. Real-time quantitative polymerase chain reaction (RT-qPCR) was used to test miRNA expression levels (TaqMan™ Fast advanced master mix by Thermo Fisher Scientific, Waltham, MA, USA). All miRNA results were normalized to a housekeeping miRNA miR-191-5p.

### 2.4. mRNA and miRNA Profiling

Transcriptome profiling was performed by Genome Quebec (Montreal, Canada) using the Illumina Whole-Genome II Expression BeadChips (MouseRef-8 v2.0) GeneChip Mouse Genome Array (San Diego, CA, USA). Illumina small RNA protocol (Illumina, Inc., San Diego, CA, USA) was used to prepare miRNAs libraries at the Department of Molecular and Cell Biology, Baylor College of Medicine, Houston. Sequencing was completed using the Illumina Genome Analyzer NextSeq 500 (Illumina, Inc., San Diego, CA, USA).

### 2.5. Quality Control, Mapping and Assembly

The quality of raw sequence reads was checked using FastQC v0.10.1 High Throughput Sequence QC Report (Version 0.11.2) [30] (Babraham Institute, Cambridge, UK). The reads from Agt Tg and Wt mice were mapped to the Mus musculus genome GRCm38.p4 (NCBI mouse genome build 10) using Qseq^®^ software version 12 (DNASTAR Madison, WI). The Gunaratne Next Generation Pipeline was used to extract miRNA expression profiles [31]. Raw data were analyzed by R Bioconductor package lumi [32] followed by linear modeling framework in Bioconductor R Package limma for assessing differentially expressed genes with *p*-value < 0.05 and absolute fold change >1 [33]. miRNA sequencing reads were mapped against the miRbase [34] reference onto miRNA precursors, and reads mapped to mature miRNAs were selected.

### 2.6. Data Availability

Microarray and small RNA-Seq data for Agt Tg and Wt mice were submitted to Gene Expression Omnibus (GEO) data repository (http://www.ncbi.nlm.nih.gov/projects/geo/). Agt Tg and Wt microarray and Small RNA-Seq data were approved under accession number GSE147935 and GSE149231, respectively.

### 2.7. Differential Expression Analysis and Prediction of miRNA-mRNA Functional Interactions

We used R Bioconductor, package limma, to identify the differentially expressed genes and miRNAs. We calculated fold change with respect to the Agt Tg mice (i.e., the upregulated genes/miRNAs are the genes/miRNAs with expression of the Agt Tg mice samples that are higher than the expression of these genes/miRNAs in the Wt mice). Package anamiR was used to integrate and analyze mRNA data and miRNA data [35]. Further, predicted algorithm TargetScan (release 7.2; http://www.targetscan.org/) was used to identify predicted targets of miRNAs and vice versa [36]. Additionally, other algorithms such as miRecords [37], DIANA-microT-CDS [38] and EIMMo [39], were used to identify target–miRNA interactions. Selected possible targets as well as miRNAs were validated in both WAT from Agt Tg and Wt mice and in 3T3-L1 cells treated with or without Ang II.

### 2.8. Cell Transfections (Using miRNAs Mimics and Inhibitors)

miR-690 mimic, inhibitor along with miRNA negative control/scramble (NC) (Applied Biological Materials, British Columbia, Canada) were used. Three different concentrations of miR-690 mimic, inhibitor and NC (20 nM, 40 nM, 80 nM) were used to identify optimal concentration. Differentiated 3T3-L1 adipocytes were transfected according to the manufacturer’s protocol in 6-well plates using lipofectamine RNAi-max for 48 h. (Thermo Fisher Scientific, Waltham, MA, USA). Following transfection, cells and media were collected.

### 2.9. Cloning, Construct Preparation and Luciferase Assay

Specific forward and reverse primers (using NEBuilder assembly tool) were used to synthesize a 400–300 base pair fragment of 3′ untranslated regions (UTRs) for each target gene including binding sequence for miR-690. Synthesized 3′ UTR fragments were amplified by PCR using mouse DNA as a template. The PCR products were subcloned into PSICheck2 vector (Promega, Madison, WI, USA) at Xho1 and Not1 sites downstream of the Renilla luciferase reporter gene using Gibson assembly (New England Biolabs, Ipswich, MA, USA). Constructs were then transformed into *Escherichia coli* competent cells (Promega, Madison, WI, USA) and screened with ampicillin resistance plates. Colony PCR was performed using PSICheck2 forward and reverse primer for the 3′ UTR of the target genes to confirm cloning accuracy. Following this, sequence was verified to confirm that there were no mutations on the cloned section (at the binding sequence for miR-690) before performing luciferase assay.

3T3-L1 preadipocytes were plated in 96-well plates. One hundred nanograms of DNA constructs (PSICheck2 vector with 3′ UTRs fragments of target genes) along with 40 nM miR-690 mimic or 40 nM miRNA NC were co-transfected into 3T3-L1 cells using Lipofectamine 3000 (Thermo Fisher Scientific, Waltham, MA, USA). Luciferase activity was measured 48 h post-transfection with the Dual Luciferase Reporter Assay System (Promega, Madison, WI, USA) as per the manufacturer’s protocol. Luciferase activity was reported as relative luciferase units (firefly luciferase/Renilla luciferase). Based on the luciferase assay results, miR-690 binding sequence on 3′UTR of MAP2K3 was mutated (with “ATAAAAA”) using Q5^®^ Site-Directed Mutagenesis kit (New England biolabs, Ipswich, MA, USA) and mutants were sequence verified. 3T3-L1 cells were co-transfected with MAP2K3 construct and mutated MAP2K3 construct with miR-690 or NC followed by Luciferase assay as described previously.

### 2.10. Western Blotting and ELISA

Bradford protein assay was used to measure total protein concentration (Bio-Rad, Hercules, CA, USA). The separation of whole protein lysates was performed using SDS-PAGE and transferred to polyvinylidene difluoride (PVDF) membranes (Millipore, Burlington, MA, USA). Following this, blots were incubated with primary antibodies such as tubulin (Sigma-Aldrich, St. Louis, MO, USA), p38, p65 (Cell Signaling Technology, Danvers, MA USA), GRP78 (BiP) and Gadd 153 (CHOP) (Santa Cruz Biotechnology, Dallas, TX, USA) were used [40]. One-hour incubation of membranes with anti-mouse or rabbit secondary antibodies was (Jackson Immuno Research Laboratories, West Grove, PA, USA) conducted before detecting fluorescence using LI-COR Odyssey machine (LI-COR Odyssey CLX, Lincoln, NE, USA).

Enzyme-linked immunosorbent assay (ELISA) kits (R&D Minneapolis, MN, USA) were used to measure IL-6 levels in the supernatants according to the manufacturer’s protocol.

### 2.11. Statistical Analyses

All results were presented as means ± SEM, and one-way ANOVA was used with Tukey’s post-hoc test (*p* < 0.05) for experiments with three or more groups. On the other hand, Student’s t test was used to analyze data with two groups. CFX Manager software provided by Bio-Rad Laboratories, Inc. and QuantStudioTM design and analysis software v1.5.1 were used to analyze qRT-PCR assays. All mouse experiments had 6–8 replicates, while in vitro cell experiments had 3–5 replicates.

## 3. Results

Mice with Agt overexpression (Agt Tg) showed an obese phenotype characterized by adipocyte hypertrophy and increased ER stress and adipose inflammation [2,3]. However, exact molecular pathways and miRNAs involved in the upregulation of cell stresses and inflammation with RAS overactivation remain unclear.

### 3.1. Genes and miRNAs are Differentially Expressed in White Adipose Tissue (WAT) When Renin Angiotensin System (RAS) is Overexpressed

We used WAT from mice overexpressing Agt in the adipose tissue (Agt Tg) to identify differentially expressed genes and miRNAs. A total of 9459 genes were expressed in Agt Tg and Wt groups, out of which 895 genes were differentially expressed (based on absolute fold change ≥1 and *p*-value ≤0.05). Between the two groups, 338 and 557 genes were up- and downregulated in Agt compared to Wt mice, respectively. Micro-array results were then analyzed by Wiki-pathways [41] to identify top pathways affected by RAS overactivation. There were several metabolic pathways significantly upregulated in Agt Tg mice compared to Wt mice. These include several inflammatory, oxidative-stress and p38 MAPK-signaling pathways, and the top 10 significant pathways (*p* < 0.05) are listed in Table 1.

**Figure 1:**
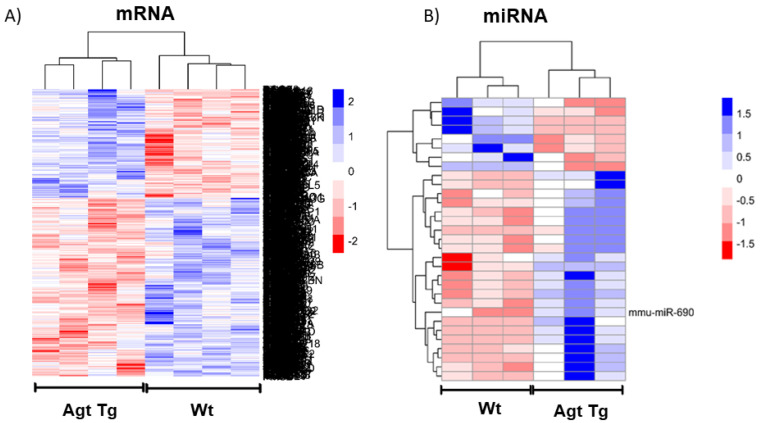
Profiling of genes and miRNAs in epididymal white adipose tissue (WAT) of mice overexpressing angiotensinogen (Agt Tg) compared to wild-type (Wt) mice. (**A**) Heat map representation of transcripts overexpressed (blue) and underexpressed (red) in Agt Tg vs. Wt mice WAT (*p* < 0.05, fold change >2). Rows represent transcripts while columns are samples. (**B**) Heat map representation of miRNAs overexpressed (blue) and underexpressed (red) in Agt Tg vs. wild-type mice WAT (*p* < 0.05, fold change >2). Rows indicate differentially expressed miRNAs, and columns are profiled samples. One candidate miRNA, mir-690, is labeled, others not shown. (mRNA expression: *n* = 4 each group, miRNA expression: *n* = 3 each group).

A similar approach was used to identify 31 differentially expressed miRNAs (based on absolute fold change ≥1 and *p*-value ≤0.05) in WAT, out of which 23 and 8 miRNAs were upregulated and downregulated, respectively, in Agt Tg compared with Wt mice (Figure 1B). Differential expression of miRNAs and genes between Agt Tg and Wt mice were analyzed through hierarchical clustering (Figure 1A,B).

### 3.2. Validation of miR-690 in White Adipose Tissue (WAT) and Identification of Potential Target Genes of miR-690

**Figure 2:**
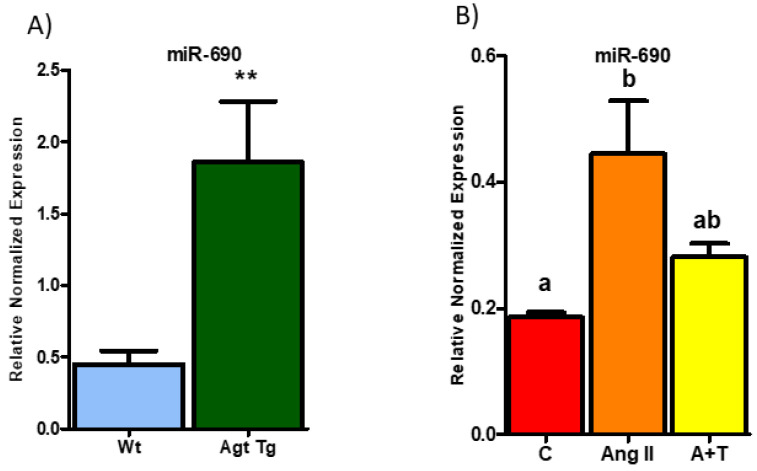
Renin–angiotensin system (RAS) overexpression induces miR-690 expression in both epididymal white adipose tissue (WAT) and adipocytes. (**A**) miR-690 expression is significantly higher (3-fold) in WAT in mice overexpressing angiotensinogen (Agt Tg) compared to wild type (Wt) mice (** *p* < 0.001) (*n* = 8). (**B**) Angiotensin treatment in 3T3-L1 adipocytes significantly increased miR-690 levels compared to control group, and miR-690 expression was reduced with RAS inhibitor telmisartan (trending with *p*-value 0.09) compared Ang II treated group (C = control, A = Ang II, A+T = Ang II + Telmisartan). Common letters on the error bars indicate no significance (e.g., “a” is significantly different from “b” and “ab” indicates no significance compared to “a” and “b”). Data is presented as mean ± SEM (*p* < 0.05). (*n* = 4).

Out of 31 differentially regulated miRNAs, we selected miR-690 as the top candidate for further study for the following reasons. miR-690 was significantly upregulated by ~2.9-fold in Agt Tg compared to Wt mice in sequencing data (Figure 1B). As expected, Agt Tg mice showed 3-fold higher (*p* < 0.001) miR-690 expression in WAT compared to Wt mice (Figure 2A). Furthermore, 3T3-L1 cells treated with Ang II had 2-fold higher expression of miR-690 compared to control (Figure 2B). To further confirm the involvement of RAS in upregulating miR-690 expression, we treated 3T3-L1 cells with telmisartan (Ang II type 1 (AT1) receptor blocker), and miR-690 expression was considerably downregulated compared to the Ang II-treated group (Figure 2B).

To determine the potential regulatory role of miR-690, micro-array results were integrated with miRNA sequencing results to identify miR-690 target genes. Based on the results shown in Table 2, miR-690 was predicted to regulate several pathways related to inflammation, cell stress and several genes including MAPKs (Table 2). Hence, to elucidate whether miR-690 directly regulates genes in above pathways, potential interactions were tested using target prediction algorithms TargetScan.

### 3.3. Gene Validation of Predicted Targets of miR-690 in White Adipose Tissue (WAT) and Adipocytes

**Figure 3:**
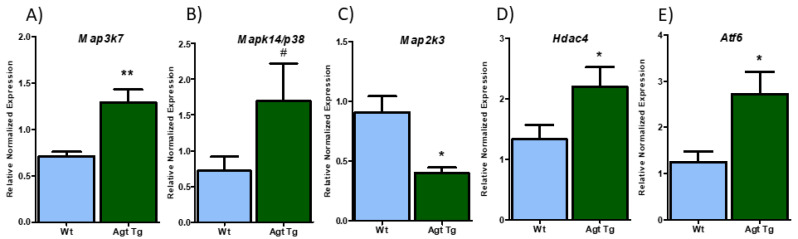
Renin–angiotensin system (RAS) overexpression differentially regulates genes related to inflammation and cell stress in mice epididymal white adipose tissue (WAT). (**A**,**B**) RAS overexpression induces mitogen-activated protein kinase kinase kinase 7 (*Map3k7*) and mitogen activated protein kinase 14 (*Mapk14/p38*) expression compared to wild type (Wt) mice. (**C**) However, RAS overexpression significantly downregulated mitogen activated protein kinase kinase 3 (*Map2k3*) expression in Agt Tg compared to Wt mice. (**D**,**E**) Similar to *Map3k7*, histone deacetylase 4 (*Hdac4*) and activating transcription factor 6 (*Atf6*) were upregulated in Agt Tg group compared wild-type (Wt) mice WAT. Data are presented as mean ± SEM (*n* = 8). (** *p* < 0.001, * *p* < 0.05, # *p* < 0.1).

**Figure 4:**
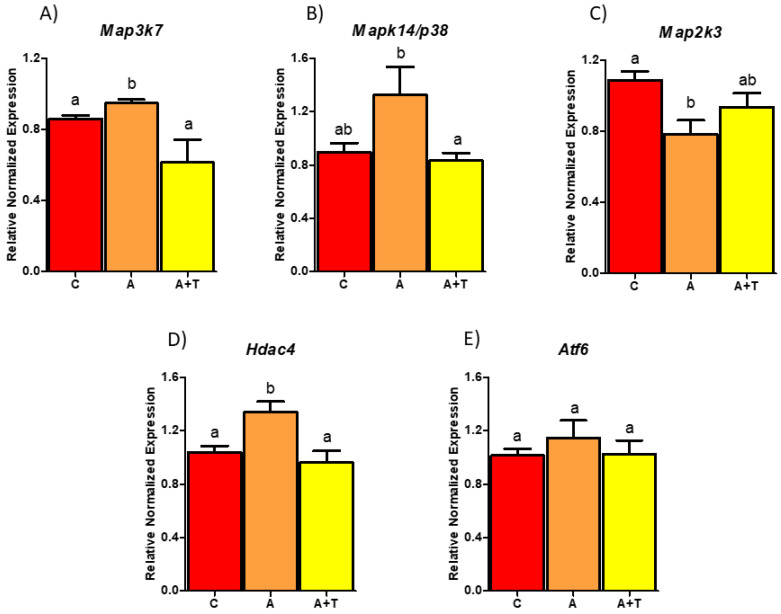
Renin–angiotensin system (RAS) overexpression differentially regulates genes related to inflammation and cell stress in 3T3-L1 adipocytes. (**A**,**B**) RAS overactivation induces mitogen-activated protein kinase kinase kinase 7 (*Map3k7*), mitogen-activated protein kinase 14 (*Mapk14/p38*) in Ang II treated group compared to control group, whereas Ang II treatment along with telmisartan significantly reduced gene expression of respective markers. (**C**) RAS overactivation significantly downregulated mitogen activated protein kinase kinase 3 (*Map2k3*) expression in Ang II treated group compared control group. (**D**) Meanwhile, histone deacetylase 4 (*Hdac4*) showed a similar expression as observed in *Map3k7* (**E**) No changes were observed in activating transcription factor 6 (*Atf6*) expression with Ang II or telmisartan in 3T3-L1 cells. (C = control, A = Ang II, A+T = Ang II + Telmisartan). Common letters on the error bars indicate no significance (e.g., “a” is significantly different from “b” and “ab” indicates no significance compared to “a” and “b”). Data are presented as mean ± SEM (*p* < 0.05). (*n* = 5).

Several MAPKs with predicted binding site for miR-690 were identified and initially validated in WAT from Agt Tg and Wt mice. Higher expression of mitogen-activated protein kinase kinase kinase 7 (*Map3k7*) (*p*-value = 0.001) and *Mapk14* (*p*-value = 0.058) was observed in Agt Tg mice (Figure 3A,B); however, *Map2k3* showed significantly reduced expression in Agt Tg compared to Wt mice (Figure 3C). To further verify the potential regulatory role of miR-690 on metabolic pathways, oxidative stress marker *Hdac4* and ER stress associated gene activating transcription factor 6 (*Atf6*), both predicted to be regulated by miR-690, were validated. Both *Hdac4* and *Atf6* showed significantly higher mRNA levels in Agt Tg compared to Wt mice (Figure 3D,E). These predicted targets were then validated in clonal mice 3T3-L1 adipocytes. Cells treated with Ang II demonstrated significantly upregulated expression of *Map3k7*, *Mapk14* and *Hdac4* compared to control group, whereas AT1 antagonist telmisartan significantly reduced Ang II-induced gene expression of *Map3k7*, *Mapk14* and *Hdac4* (Figure 4A,B,D). On the other hand, *Map2k3* was significantly reduced with Ang II treatment compared to control group; however, telmisartan did not fully recover Ang II effects, although it was trending (*p*-value of 0.192) (Figure 4C). Moreover, *Atf6* did not show significant changes in cells with RAS overactivation, even though we observed changes in mice WAT (Figure 4E).

### 3.4. Regulatory Effect of miR-690 on Potential Target Genes

**Figure 5:**
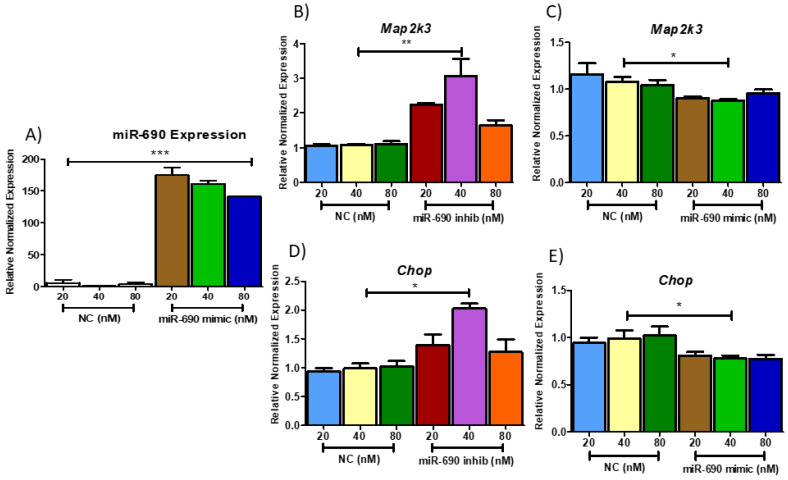
Regulatory role of different concentrations of miR-690 on its targets. (**A**) miR-690 transfection validation. All 3 different concentrations (20–80 nM) of miR-690 mimic-treated groups showed significantly higher miR-690 expression compared to negative controls (NC). (**B**) Potential target of miR-690, mitogen activated protein kinase kinase 3 (*Map2k3*) showed significantly higher expression with 40nM inhibitor compared to NC groups. (**C**) The 40 nM mimic concentration showed significantly reduced *Map2k3* expression compared to 40 nM NC. (**D**) C/EBP homologous protein (*Chop*) showed significantly higher expression with 40nM inhibitor compared to NC groups and (**E**) 40 nM mimic concentration showed significantly reduced *Chop* expression compared to 40 nM NC. Data are presented as mean ± SEM (***p < 0.0001, ** *p* <0.001, *p < 0.05). (*n* = 3).

Mouse adipocytes were treated with three different concentrations (20, 40 and 80 nM) of miR-690 mimic, inhibitor and NC to identify the optimal concentration. Transfection efficiency was initially tested by analyzing the expression of miR-690 in mimic- and NC-treated cells. As anticipated, 150-fold higher miRNA expression was observed in miR-690 mimic-treated cells compared to NC, confirming that transfection was successful (Figure 5A). Following this, *Map2k3*, and C/EBP homologous protein (*Chop*) expressions were tested, and a 40 nM concentration of miR-690 inhibitor showed significantly higher expression for both *Map2k3* and *Chop* compared to NC groups (Figure 5B,D), while significantly reduced *Map2k3/Chop* levels were indicated in 40 nM mimic-treated groups (Figure 5C,E). Therefore, cells were treated with 40 nM concentration of miR-690 mimic, inhibitor, NC (48 h transfection) for future experiments and mRNA levels of predicted miR-690 targets were tested.

**Figure 6:**
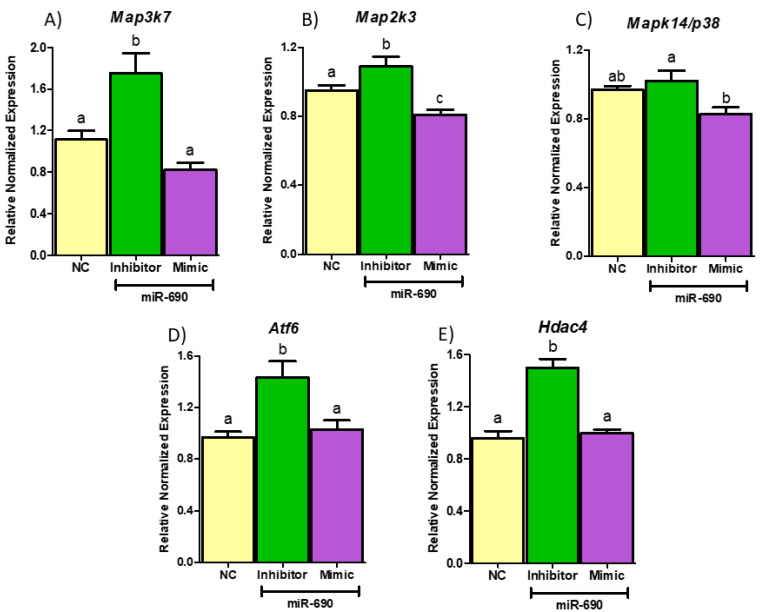
miR-690 target validation with miRNA mimic and inhibitor treatments. (**A**,**B**) miR-690 inhibitor significantly induced expression of mitogen activated protein kinase kinase kinase 7 (*Map3k7*) and mitogen activated protein kinase kinase 3 (*Map2k3*) expression, while, as expected, miR-690 mimic significantly reduced their expression compared to negative control (NC). (**C**) No significant changes were observed with miR-690 mimic or inhibitor treatments in mitogen activated protein kinase 14 (*Mapk14/p38*) expression in 3T3-L1 cells compared to NC groups. (**D**,**E**) Only miR-690 inhibitor was able to increase expression of histone deacetylase 4 (*Hdac4*) and activating transcription factor 6 (*Atf6*) expression but not mimic compared NC. Common letters on the error bars indicate no significance (e.g., “a” is significantly different from “b” and “ab” indicates no significance compared to “a” and “b”). Data are presented as mean ± SEM (*p* < 0.05). (*n* = 4).

To confirm the regulatory effects of miR-690 on selected genes (*Map2k3, Map3k7, Mapk14, Hdac4, Atf6*), differentiated 3T3-L1 adipocytes were transfected with miR-690 mimic, inhibitor and NC. As indicated in Figure 6, miR-690 inhibitor significantly upregulated expression of *Map3k7* and *Map2k3* compared to their respective NC groups (Figure 6A,B). In contrast, miR-690 mimic-treated groups showed significantly reduced *Map3k7* and *Map2k3* expression (Figure 6A,B). Another MAPKs, *Mapk14/p38,* did not show significant changes with either miR-690 mimic or inhibitor treatments compared to NC, although it was identified as a possible target of miR-690 from bioinformatic analysis. However, *Hdac4* and *Atf6* exhibited significantly high mRNA levels in miR-690 inhibitor treated groups *(p <* 0.05); however, miR-690 mimic did not show the expected reduction compared to the NC group (Figure 6D,E).

### 3.5. Mouse miR-690 Targets MAPKs to Regulate Downstream Signaling Pathways

**Figure 7:**
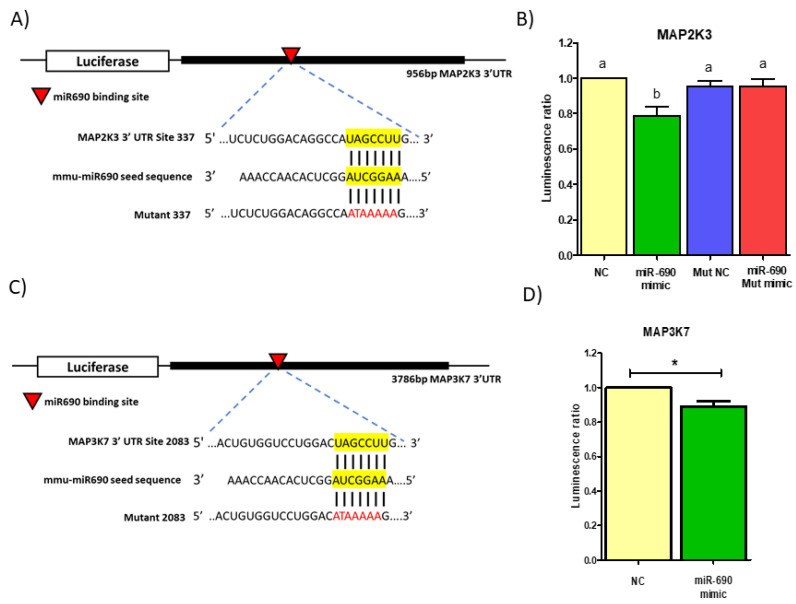
miR-690 directly regulates mitogen activated protein kinase kinase 3 (*Map2k3*) expression via binding to 3′ untranslated region (3′UTR). (**A**) Representation *Map2k3* with miR-690 at 3′UTR on the reporter plasmid. Predicted binding sites (using computational tools) for miR-690 in the *Map2k3* 3′UTR are indicated with red arrowhead. Complementary seed region for miR-690 was mutated to block miRNA target binding (mutated nucleotides are indicated in red). (**B**) Luciferase reporter activity of the *Map2k3* 3′UTR in 3T3-L1 cells treated with miR-690 mimic and negative control (NC). Reporter activity decreases by 20% when cells are treated with mimic. Moreover, mimic did not reduce luciferase activity in mutated 3′UTR group compared to NC. (**C**) Representation of mitogen activated protein kinase kinase kinase 7 (*Map3k7*) by miR-690 at 3′UTR on the reporter plasmid and the predicted binding sites for miR-690 in the *Map3k7* 3′UTR are in red arrowhead. (**D**) Luciferase reporter activity of the *Map3k7* 3′UTR in 3T3-L1 cells treated with miR-690mimic and NC. Reporter activity decreased only by 10%. Each sample was normalized to Renilla luciferase activity. Common letters on the error bars indicate no significance (e.g., “a” is significantly different from “b”). Data are presented as mean ± SEM (*p < 0.05). (*n* = 4).

To determine whether miR-690 directly regulates the expression of target genes via 3′UTR binding, we cloned a segment of 3′UTR containing the miR-690 binding sequence of respective targets into a luciferase reporter. Out of the five targets we selected, only *Map2k3* and *Map3k7* were used for luciferase reporter assays as they displayed best results with miR-690 mimic and inhibitor treatments. Both *Map2k3* and *Map3k7* have a single potential binding site on 3′UTR at position 337-343 and 2083-2089, respectively (Figure 7A,C). Differentiated 3T3-L1 cells were co-transfected with cloned PsiCheck2vector, miR-690 mimic and NC. Based on luciferase assay results, miR-690 mimic-treated group showed significantly reduced luciferase activity compared to NC for *Map2k3* (~20% reduction) as well as *Map3k7* (~10% reduction) (Figure 7B,D). *Map2k3* was selected for further analysis as it showed significantly better reduced luciferase activity compared to *Map3k7*. To further confirm direct inhibitor role of miR-690, miRNA binding site on the 3′UTR of *Map2k3* was mutated via site-directed mutagenesis and luciferase activity was measured. When putative binding site on 3′UTR was mutated, inhibitory activity of miR-690 on *Map2k3* was abolished as shown by recovered luciferase activity in Figure 7B (groups NC mut and Mimic mut). These results confirmed the direct regulatory role of miR-690 on *Map2k3* expression.

**Figure 8:**
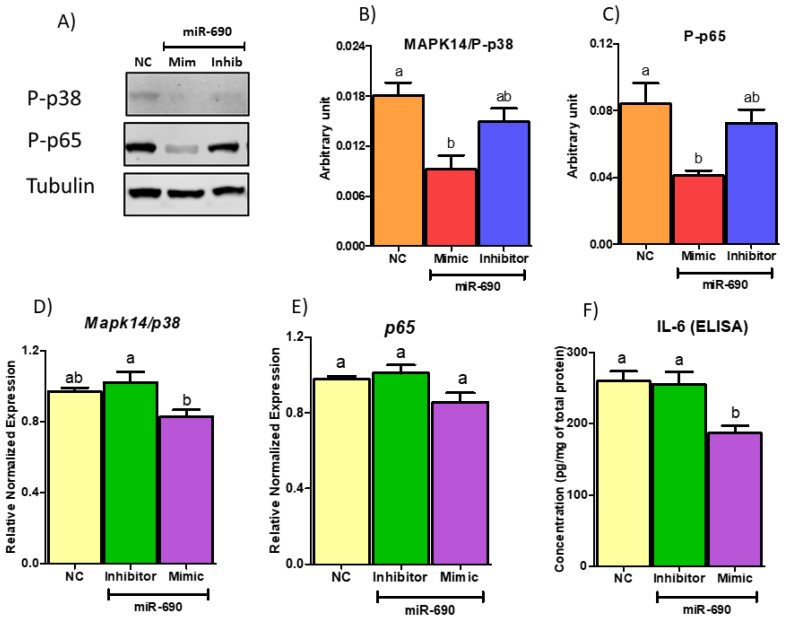
Targeting mitogen activated protein kinase kinase 3 (*Map2k3*) by miR-690 reduces downstream inflammation and cell stress. (**A**) Western blot image of protein analysis. miR-690 mimic significantly reduced the amount of phospho-p38 (P-p38) and phospho-p65 (P-p65) at protein level compared to negative control (NC), whereas inhibitor-treated groups had no differences in the protein amount compared to NC. (**B**) Quantification of P-p38 protein normalized to Tubulin. (**C**) Graphical interpretation of P-p65 protein level normalized to Tubulin level. (**D**) *Mapk14/p38* did not show any significant reduction in messenger RNA (mRNA) level with miR-690 mimic or inhibitor treatments compared to NC. (**E**) Similar to *p38, p65* showed no changes at mRNA levels among treatment groups. (**F**) Enzyme-linked immunosorbent assay (ELISA) results. miR-690 mimic significantly reduced interleukin 6 (IL-6) level in media treated with miR-690 mimic compared to NC. Common letters on the error bars indicate no significance (e.g., “a” is significantly different from “b” and “ab” indicates no significance compared to “a” and “b”). Data are presented as mean ± SEM (*p* < 0.05) (*n* = 4).

**Figure 9:**
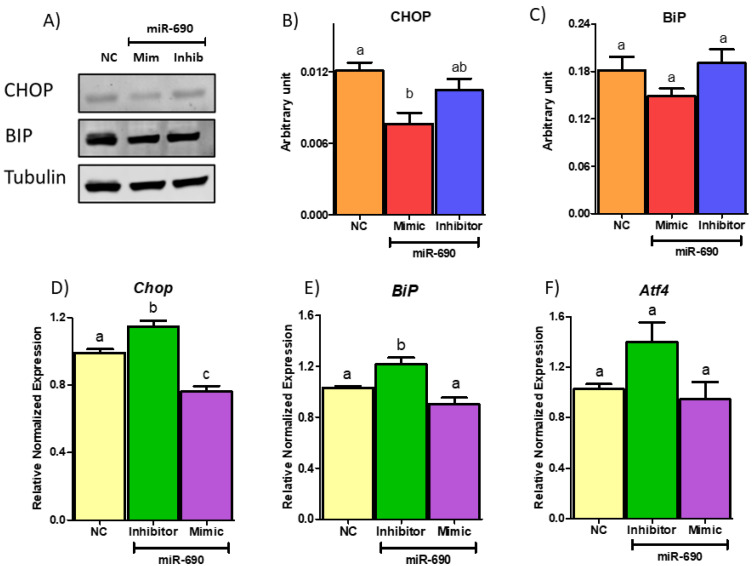
Effects of targeting mitogen-activated protein kinase kinase 3 (*Map2k3*) by miR-690 on ER stress markers. (**A**) Western blot image of protein amounts of C/EBP homologous protein (CHOP) and binding immunoglobulin protein (BIP). Mir-690 Mimic significantly reduced CHOP expression compared to negative control (NC) group, but no changes were observed with inhibitor. BIP protein amounts were unchanged among NC, miR-690 mimic and inhibitor groups. (**B**) Quantification of CHOP protein normalized to Tubulin. (**C**) Quantification of BIP protein normalized to Tubulin. (**D**) Gene expression of *Chop*. miR-690 inhibitor significantly induced *Chop* expression, whereas mimic treatment reduced *Chop* expression as expected compared to NC group. (**E**) miR-690 inhibitor treated group had significantly increased mRNA level of *BiP* compared to NC group, yet no changes were observed among mimic and NC treated groups (**F**) No differences were observed in activating transcription factor 4 (*Aft4*) gene expression with treatments of miR-690 inhibitor or mimic compared to NC group. Common letters on the error bars indicate no significance (e.g., “a” is significantly different from “b” and “ab” indicates no significance compared to “a” and “b”). Data are presented as mean ± SEM (*p* < 0.05). (*n* = 4).

To investigate whether miR-690 regulates inflammation and ER stress via *Map2k3* axis, we tested several downstream targets at RNA and protein level. Since *Map2k3* regulates inflammatory and cell stress pathways by phosphorylated activation of *Mapk14/p38*, we initially tested the expression of p38 at both gene and protein level in adipocytes treated with NC, miR-690 mimic and inhibitor (co-transfected for 48 h). p38 and p65 production was reduced by miR-690 mimic treatment compared to NC at protein level (Figure 8A–C). However, miR-690 inhibitor did not show changes in protein level compared to both mimic and NC groups (Figure 8A–C). Gene expression analysis was followed by protein experiments, and no significant differences were observed between NC/inhibitor or NC/mimic for p38, yet mRNA levels were significantly different among miR-690 inhibitor and mimic-treated groups (Figure 8D). To further understand the effects of miR-690 on inflammation, the mRNA level of NF-κB subunit p65 was measured. Although there were significant changes at protein level for p65, no changes were observed at mRNA levels among treatments (Figure 8E). Corroborating with p65 protein data, ELISA results indicated a significant reduction of IL-6 protein level in miR-690 mimic-treated media compared to NC, but no differences were observed in inhibitor group compared to NC (Figure 8F). Finally, to demonstrate the potential effects of miR-690 on ER stress, we tested a few ER stress markers such as CHOP and binding immunoglobulin protein (BIP). Protein amount of CHOP was significantly reduced by miR-690 mimic-treated group compared to NC group (Figure 9A,B). However, no changes were observed with miR-690 inhibitor (Figure 9A,B). Another ER stress marker BIP did not show any differences among three groups, although the mimic-treated group showed a reducing trend with *p* = 0.087 (Figure 9A–C). Interestingly, *Chop* and *BiP* showed a significantly higher expression with inhibitor compared to NC-treated group at mRNA level (Figure 9D,E). Significantly lower gene expression was only observed in *Chop* with mimic treatment compared to NC-treated group; however, mimic was unable to reduce the expression of *BiP*, although it was trending in the correct direction compared to NC (*p*-value = 0.0506) (Figure 9D,E). Activating transcription factor 4 (*Atf4*) is another ER stress marker which further activates *Chop*, and it did not show changes with either miR-690 mimic or inhibitor compared to the NC group (Figure 9F). These results indicate that miR-690 may be used as a target since it shows the potential ability to reduce inflammatory and ER stress pathways directly targeting *Map2k3*.

## 4. Discussion

Ang II is the main effector peptide of RAS and one of the major pro-inflammatory adipokines produced by obese adipose tissue. Abnormalities of adipose RAS secretion could directly disrupt metabolic homeostasis leading to obesity and related chronic diseases [42,43]. Although RAS components are overexpressed in adipose tissue during obesity, mechanisms by which RAS disrupts cellular homeostasis are not well understood. To dissect molecular mechanisms, we performed global gene and miRNA profiling using WAT samples from Agt Tg and Wt mice. We identified 31 differentially expressed miRNAs and numerous signaling pathways (inflammatory pathways, MAPK Signaling Pathway, oxidative stress) upregulated with RAS overexpression in WAT (Figure 1B and Table 1).

Several lines of evidence show that RAS expression is widely regulated by miRNAs [15,44,45,46,47] and vice versa [15,16,18,48]. However, no studies have been performed specifically in adipose tissue to determine the role of miRNAs in RAS-linked obesity. Since adipose RAS contributes to approximately one-third of total systemic RAS production, identifying potential miRNAs and miRNA-regulated target genes which are affected by RAS overactivation could be used as probable therapeutic targets to alleviate RAS-associated metabolic disorders. The role of miRNAs may differ depending on the context and cellular milieu [49,50,51]. In some situations, higher activity of a miRNA cause detrimental effects and are involved in the progression of diseases [52]. In other conditions, miRNAs play a beneficial role by providing a protective function [48]. In the current study, we showed for the first time that miR-690 provides a protective function against RAS-induced ER stress and inflammation by targeting MAP2K3 in WAT. Confirming miRNA profiling results, miR-690 was significantly upregulated with RAS in both in vivo and in vitro experiments (Figure 2A,B), and computational analysis further confirmed the potential ability of miR-690 to impact MAPK signaling through several predicted targets (TargetScan) including MAPK14, MAP2K3 and MAP3K7.

Among genes which possess complementary binding site for miR-690 (in the 3′UTR), we identified MAP2K3, a key component of p38MAPK signaling pathway, as a direct target of miR-690. A typical MAPK signaling pathway comprises three different kinases (upstream Ser/Thr kinase, middle dual-specificity kinases and downstream Ser/Thr kinase) [53]. MAP3K7 is one of the upmost kinases of p38MAPK signaling pathway where it directly stimulates MAP2Ks (e.g., MAP2K3, MAP2K6) [54]. Activation of MAP2Ks further phosphorylates p38 kinases (MAPK14), a known regulator of several downstream pathways associated with inflammation and cellular stresses. Induction of p38 kinases (p38MAPKα (MAPK14), p38MAPKγ and p38MAPKδ) by MAP2K3 is directly involved in inflammation by provoking IL-12 [55]. Some of the other pro-inflammatory cytokines activated by p38MAPK are TNFα, NF-κB, IL-6 and IL-1 [56,57,58,59]. Interestingly, Ang II is involved in rapid activation of p38 via phosphorylation in vascular smooth muscle cells [60,61]. Corroborating with these, we demonstrated that RAS overactivation in WAT upregulates p38 and MAP3K7 expression. Additionally, research conducted for the past few decades has shown that p38 MAPK also induces ER stress [21,22,23,62]. Luo et al. reported that induction of BiP requires the involvement of p38 MAPK signaling in adipocytes [22]. Moreover, p38 activates CHOP, bypassing ER stress related to UPRs via direct phosphorylation [21]. In our previous study, we revealed that adipose RAS overexpression drastically activated ER stress and inflammation [3] via an unknown pathway. In the current study, we demonstrate that RAS-associated ER stress and inflammation could be potentially mediated via activating p38MAPK signaling axis. Therefore, selective regulation of p38MAPK signaling by directly targeting p38 (*Mapk14*) or upstream targets such as MAP2K3/MAP3K7 would be a potential approach to reduce RAS induced metabolic alterations.

Using miR-690 mimic and inhibitors, we confirmed that miR-690 directly targets and regulates MAP2K3 expression by binding to complementary site at 337-343 position on 3′UTR of MAP2K3. Interestingly, miR-690 mimic reduced MAP2K3 expression at mRNA levels (Figure 6B). While early studies indicated that miRNAs-mediated gene regulation is due to translational repression [63,64], subsequent studies have revealed alternate mechanisms for miRNA activity, which include degradation of mRNA targets of miRNAs [6,7,8,9]. miRNA-mediated mRNA decay (via decapping and 5′–3′ mRNA decay) contributes to lower mRNA levels, which is in line with the reduced MAP2K3 gene expression we observed. Furthermore, Eichhorn et al. showed that when miRNAs are expressed at constant levels (steady state), miRNA-mediated repression is mostly by mRNA decay (66%–90%), compared to translational repression, which is roughly about 10%–25% [65]. These results further support our in vivo and in vitro results for MAP2K3, where constant expression of miR-690 in mice by RAS overexpression (steady state) resulted in 50% reduction of MAP2K3 expression compared to 10% reduction in 3T3-L1 cells, which were incubated for a short (24 h) time with Ang II.

An interesting study performed in mice heart muscle identified by sequencing that miR-690 (along with other miRNAs) was enriched in mitochondria during early stages of failing heart [66]. Interestingly, miR-690 level was significantly increased only during early stage of heart failure but not at a later stage [66]. A higher level of miR-690 during early stages of failing heart may indicate a protective role of miR-690 to restore metabolic homeostasis in the heart. These findings are indeed consistent with our data, in which we observed higher miR-690 in adipose tissue with RAS overactivation during early stages of obesity. However, the exact role of miR-690 in failing heart needs further validation beyond its predicted functions based on bioinformatic tools. Moreover, Yu et al. reported miR-690 as a positive regulator of Runx2-induced osteogenic differentiation of C2C12 myogenic progenitor cells [29]. Conversely, in myeloid-derived suppressor cells (MDSCs: bone-marrow-derived group of immune cells), miR-690 reduced MDSCs expansion and differentiation by directly targeting CCAAT/enhancer-binding protein α (C/EBPα) [28]. Similarly, it is possible that miR-690 may reduce adipose differentiation and play an important regulatory role in adipose functions. Proving the above predictions, we demonstrated the involvement of miR-690 in regulating inflammation and ER stress in adipocytes. Our results confirmed that miR-690 is capable of reducing inflammation (e.g., NF-κB and IL-6) and ER stress markers (e.g., CHOP) in clonal mouse adipocytes, potentially by targeting MAP2K3. Hence, significantly high miR-690 expression may be a positive feedback mechanism to attenuate RAS-induced inflammation and ER stress. Biological systems and metabolic pathways are regulated by internal and external stimuli. However, if these stimuli are involved in disrupting cellular homeostasis (due to prolong or chronic activation/downregulation of signaling pathways), biological systems are naturally designed to activate protective mechanisms to alleviate harmful outcomes. For instance, increased IL-10 levels (anti-inflammatory cytokine) during infections and lung inflammation provides protection against detrimental effects [67]. In line with this, our observation of induced miR-690 expression due to RAS overactivation could be a potential defensive process to indirectly regulate destructive involvement of RAS overexpression. A similar protective function of a miRNA was demonstrated in a recent study conducted by He et al. where they showed that miR-21 provides a protective role against coxsackievirus B3 infection by targeting MAP2K3 [68]. Thus, targeting MAP2K3 by miR-690 could be used as a potential remedial approach to mitigate RAS associated metabolic disruptions.

Interestingly, miR-690 mimic did not lower levels some of the predicted targets (MAPK14, HDAC4 and ATF6) compared to the NC group. Although MAPK14, HDAC4 and ATF6 possess complementary binding sites in 3′UTR, miR-690 may not have actually bound with their mRNAs to regulate these genes. This may be due to the fact that these targets are false positives from TargetScan or that secondary structures near miRNA binding site at 3′UTR of mRNA. If the target site is not accessible, miRNA will not be able to regulate gene expression [69,70], and this could be the reason why we did not observe expected reduced expression with miR-690 mimic treatment for the above genes. Additionally, the plasmid vector used (psicheck2) has a strong promotor (T7) [71,72], and it may mask the luciferase activity if the miRNA mimic/inhibitor effect is smaller. This could be a reason why we observed relatively smaller or no differences in the luciferase activity for some of the predicted targets of miR-690 (e.g., MAP3K7: Figure 7D). Moreover, miR-690 could have multiple targets, where it may have more affinity towards some targets and less affinity for others. miRNA functions may also differ across tissues as there could be several other factors affecting miRNA functions. These possibilities may explain why miR-690 did not show the expected regulatory role on some of the predicted targets in this study.

Although this study fills important knowledge gaps in the literature, a few limitations exist. Changes in luciferase reporter with miRNA mimics and inhibitors are smaller than expected, possibly due to using a strong promotor [73], which may mask differences. Additionally, the promoter (aP2) we used to overexpress Agt, in the adipose tissue is also expressed in macrophages [74]. Whether the RAS overexpression in macrophages also contributed to inflammation requires further investigation. Moreover, as Ang II is also known to regulate inflammasomes by activating NLRP3, IL-1β and IL-18 in other cell types, similar mechanisms in adipose tissue are worth investigating [75,76,77]. Furthermore, reduced inflammation and ER stress by miR-690 (targeting MAP2K3) was demonstrated in in vitro studies, but whether miR-690 provides similar protective functions in vivo needs to be confirmed. Performing additional mice studies with miR-690 expression or to overexpress RAS in adipose tissue-specific manner, using adiponectin promoter may help better delineate RAS functions in metabolic tissues. Lastly, transfections (with negative controls, mimics and inhibitors) dampened the response of 3T3 L1 adipocytes to Ang II. Hence, it was difficult to perform simultaneous Ang II treatments and miR-690 transfections.

In conclusion, adipose RAS overexpression differentially expresses miRNAs and affects signaling pathways (inflammation and ER stress) which are hallmarks of obesity. This study is the first to show the effects of adipose RAS overactivation on miR-690 regulation, whereas targeted inhibition of MAP2K3 by miR-690 could be used as a novel therapeutic target to control inflammatory and cell stress signals activated via p38MAPK pathway. Increased expression of miR-690 in adipose tissue could act as a feedback mechanism to provide protection against detrimental effects of RAS overactivation. Nevertheless, future studies are needed to further dissect mechanisms linking miR-690 mediated effects (as there could be several other targets) in adipose tissue, and additional studies are warranted to validate miR-690 role in in vivo animal models, yet these investigations are beyond the scope of this manuscript.

## Figures and Tables

**Table 1:** Renin angiotensin system (RAS) overexpression affects inflammatory and cell-stress signaling pathways. Top 10 significantly different signaling pathways in Agt Tg compared to Wt mice white adipose tissue (WAT). Percentage represents the number of interested genes significantly regulated (positive genes) out of total genes included in the pathway (measured gene).

Signaling Pathway	Positive (Genes)	Measured (Genes)	Percentage (%)	Z Score	*p*-Value
IL-3	14	83	16.87	2.4	0.015
IL-6	12	72	16.67	2.18	0.028
Delta-Notch	10	53	18.87	2.42	0.015
IL-2	10	54	18.52	2.35	0.014
IL-5	9	52	17.31	2	0.024
Inflammatory Response	5	19	26.32	2.56	0.014
Oxidative Stress	5	20	25.00	2.42	0.019
Mitochondrial Gene Expression	4	16	25.00	2.17	0.032
p38 MAPK	5	24	20.83	1.95	0.047
IL-9	4	18	22.22	1.89	0.05

**Table 2:** Renin angiotensin system (RAS) mediated miR-690 activation significantly affects inflammatory and cell-stress signaling pathways. Significantly affected pathways in Agt Tg compared to Wt mice white adipose tissue (WAT) and potential gene targets of miR-690.

Signaling Pathway	Hits	Score	Expected Score	*p*-Value	Genes
IL-3	39	39	23.98	0.0049	*Atf2, Bcl2, Birc5, Bmx, Cdc42, Creb1, Crkl, Dnm1, Foxo1, Gab2, Grb2, Gsk3b, Inpp5d, Jak2, Kcnip3, Kras, Mapk1, Mapk14, Mapk7, Mapk8, Mmp2, Mras, Pak1, Pik3ca, Pik3r1, Prkca, Prkcb, Ptpn11, Rac1, Rapgef1, Rps6kb2, Rxra, Shc1, Socs2, Sos1, Src, Stat3*
IL-5	25	25	16.54	0.0533	*Atf2, Btk, Crkl, Ctnnb1, Dnm2, Foxo3, Grb2, Gsk3b, Il2rb, Jak2, Kras, Mapk1, Mapk14, Pik3cg, Pik3r1, Prkcb, Ptpn11, Rac1, Rapgef1, Rps6ka1, Sdcbp, Shc1, Shc2, Stat3, Syk*
IL-6	33	33	23.74	0.0711	*Ar, Bmx, Btk, Cd40, Crebbp, Eif4e, Erbb3, Foxo1, Foxo3, Foxo4, Gab2, Grb2, Gsk3b, Il6st, Inpp5d, Jak2, Map3k7, Mapk1, Mapk14, Mapk8, Mapt, Pik3r1, Plcg1, Ppp2r1b, Ppp2r2c, Ptpn11, Rac1, Rb1, Rps6kb1, Sgk1, Shc1, Sos1, Stat3*
IL-9	7	7	5.75	0.4644	*Grb2, Irs1, Mapk1, Pik3r1, Ptpn11, Shc1, Stat3*
Inflammatory Response	10	10	7.19	0.2825	*Cd28, Cd40, Cd80, Fn1, Il2ra, Il2rb, Lamb2, Lamc1, Lamc2, Thbs1*
Mitochondrial Gene Expression	10	10	4.55	0.0320	*Camk4, Creb1, Gabpb2, Hcfc1, Nrf1, Ppargc1a, Ppargc1b, Ppp3ca, Sp1, Tfam*
Oxidative Stress	6	6	6.71	0.5370	*Maoa, Mapk14, Mgst1, Nqo1, Sod2, Sp1*
p38 MAPK	14	14	8.63	0.0949	*Atf2, Cdc42, Creb1, Grb2, Map3k1, Map3k5, Map3k7, Map3k9, Mapk14, Mknk1, Rac1, Ripk1, Shc1, Tgfbr1*

## Supplementary Materials

The following are available online at https://www.mdpi.com/2073-4409/9/6/1327/s1, Table S1: Primer list used for mRNA quantification, Table S2. The primers used for plasmid generation.
