# Supplementary material for: Role of microRNA 690 in Mediating Angiotensin II Effects on Inflammation and Endoplasmic Reticulum Stress
Source: Cells. 2020 May 26;9(6):1327. doi: 10.3390/cells9061327 (PMC7348980; doi:10.3390/cells9061327)
Supplement: Supplementary file 1 [file cells-09-01327-s001.pdf]

Table S1. Primer list used for mRNA quantification.

Primer	Forward (5′-3′) Sequence	Reverse (5′-3′) Sequence
Mouse *Chop*	CCACCACACCTGAAAGCAGA	AGGTGAAAGGCAGGGACTCA
Mouse *Atf4*	AAGCAGCAGAGTCAGGCTTTC	GGGTTCTGTCTTCCACTCCA
Mouse *Bip*	TTCAGCCAATTATCAGCAAACTCT	TTTTCTGATGTATCCTCTTCACCAGT
Mouse *Il6*	AACCGCTATGAAGTTCCTCTC	TCCTCTGTGAAGTCTCCTCTC
Mouse *NFkB*	GGTGAAGGTCGGTGTGAAC	TGAGTGGAGTCATACTGGAACA
Mouse *Map2k3*	AGCACTTACCTACAGCCATAA	CAGAGACCGACAGGAACA
Mouse *Mapk14/p38*	AAGAGCCTGACCTATGATGAA	AGTGAAGTGAGATAGACAGAACA
Mouse *Map3k7*	ACGCTGTTGGTCTAAGGA	AGGATACTGTAACGGCTCAT
Mouse *Hdac4*	CCATTGAGAGTGAGGAGGAA	GGCTTGCTGTCTGAAGAG
Mouse *Atf6*	TGCCAAGGAGAAGATACCA	CACAGCCACAGTCACATC
Mouse *Gapdh*	CTGAGTGGAGTCATACTGGAACA	GGTGAAGGTCGGTGTGAAC
Mouse *18S*	GGACAGGATTGACAGATTGATAGC	TGCCAGAGTCTCGTTCGTTA

Table S2. The primers used for plasmid generation.

Primer	Forward (5′-3′) Sequence	Reverse (5′-3′) Sequence
psiCHECK2	CCTAACACCGAGTTCGTGAAGGT	CCCGCGTCAGACAAACCCTA
Mouse MAP2K3 UTR	aattctaggcgatcgcTCAGGGGCTCCCAGCCAG	ttttattgcggccagcCTCTCAGGAAAGTCTTAGGAGATTCATCCC
Mouse MAP2K3 mutagenesis	aaaGGTACTGTCCTGGATGCC	ttatTGGCCTGTCCAGAGAGAC
Mouse MAP3K7 UTR	aattctaggcgatcgcAGGTCTGCATCCTACAAC	ttttattgcggccagcGTAGAACAGGAATTAGTGAC
